# Mammographic density and the risk of breast cancer in Japanese women

**DOI:** 10.1038/sj.bjc.6602643

**Published:** 2005-06-14

**Authors:** C Nagata, T Matsubara, H Fujita, Y Nagao, C Shibuya, Y Kashiki, H Shimizu

**Affiliations:** 1 Department of Epidemiology & Preventive Medicine, Gifu University Graduate School of Medicine, Gifu, Japan; 2 Department of Information Culture, Nagoya Bunri University, Inazawa, Aichi, Japan; 3 Department of Intelligence Image Information, Gifu University Graduate School of Medicine, Gifu, Japan; 4 Department of Tumor and General Surgery, Gifu University Graduate School of Medicine, Gifu, Japan; 5 Gihoku General Hospital, Agata, Gifu, Japan

**Keywords:** mammography, breast cancer, case–control study, Japanese women

## Abstract

Using an automated method for detecting mammographic mass, the authors evaluated the relation between quantitatively measured density and the risk of breast cancer in a case–control study among Japanese women. The case subjects were 146 women newly diagnosed and histologically confirmed with breast cancer at a general hospital. A total of 659 control women were selected from those who attended a breast cancer mass screening at this hospital. Significantly increased odds ratios (ORs) of breast cancer were observed for breast densities of 25–49 and 50–74%, but not for densities of 75–100% as compared with 0% in premenopausal women after controlling for covariates (ORs=4.0, 4.3, and 1.4, respectively). In postmenopausal women, ORs were significantly increased for breast densities of 25–50% (OR=3.0) and 50–100% (OR=4.2). Total breast area was significantly associated with the risk of breast cancer independent of density percent or dense area in postmenopausal women. These data suggested that mammographic density was associated with the risk of breast cancer in Japanese women as is the case in Caucasian women. However, the associations of the risk of breast cancer with breast size and a high breast density greater than 75%, needs to be confirmed in future studies.

It has long been indicated that the appearance of breast parenchyma on mammograms is a marker of breast cancer risk (Wolfe, 1976). Historically, the categorical parenchymal patterns have been used to characterise breast tissue, but such classifications are subject to great variability among intra- and inter-readers (Carlile *et al*, 1983). Attempts to develop reproducible quantitative methods of assessing radiologic features of breasts began in the early 1980s. These methods have included the estimation of the percentage of mammographically detected fibro-glandular breast tissue with epithelial and connective tissue elements (as mammographic density). Harvey and Bovbjerg (2003) reviewed 12 studies reporting the association of mammographic density measured with quantitative methods to breast cancer risk (Boyd *et al*, 1982, 1995; Brisson *et al*, 1982, 1984, 1989; Wolfe *et al*, 1987; Saftlas *et al* 1991; Byrne *et al*, 1995; Kato *et al*, 1995; Van Gills *et al*, 1999; Lam *et al*, 2000; Maskarinec and Meng, 2000). All of these studies have shown moderate to strong associations of increased breast cancer risk with increased levels of mammographic density. Most of the studies have been conducted with Caucasian subjects.

The methods of measuring mammographic density are still being refined, especially with the development of digital mammography. Two of the present authors, Fujita and Matsubara, developed an automated method for detecting mammographic mass based on an adaptive thresholding technique (Matsubara *et al*, 2000). It was originally intended to serve as a computer-aided diagnosis of breast cancer. However, this method includes the detection of breast parenchyma as a process and can be easily applied to identify the dense breast area. Using this method, we conducted a case–control study to evaluate the association of mammographic breast density with breast cancer risk among Japanese women.

## MATERIALS AND METHODS

The cases for the present study consisted of incident breast cancer cases diagnosed between May 2000 and March 2002 at a general hospital in Gifu city. All cases were histologically confirmed as breast cancer. A total of 178 women agreed to participate in the study. The participation rate was 70.4%. Mammograms from the mediolateral oblique (MLO) view were not obtained from 32 cases. Therefore, the remaining 146 women comprised the cases for the present analysis.

This particular hospital has been conducting mass screening campaigns for breast cancer since the early 1980s. Women residing near the hospital were invited by the city to take part in the screening. From 2000 to 2002, the women attending the breast cancer screening at the hospital were recruited for a study of mammographic breast density. The main purpose of the study was to identify the determinants of mammographic breast density. A total of 1430 women participated in the study. The participation rate was estimated to be 70.3%. Details of the study have been described elsewhere (Nagata *et al*, 2005). From this group, 659 women who visited the screening between January 2001 and December 2002 were found to be free of breast cancer and were selected for the present study as controls. During this time period, the mammograms of both the cases and the controls were taken using identical methodologies.

Both the cases and the controls responded to a self-administered questionnaire asking basic demographic characteristics concerning smoking, physical activity, diet, drinking habits, use of medication, history of participation in breast cancer screening, medical history, and reproductive history. Menopausal status was determined by asking whether the participant had had a menstrual cycle in the past 12 months. The controls filled out the questionnaires while attending the screening. Exposure histories were recorded up to the date of diagnosis for the cases and up to the date of the screening visit for the controls. Among the cases, the median time interval between the date of diagnosis and the date that the questionnaire was filled out was 19 days. Six cases (4.1%) answered the questionnaire 3–12 months after the diagnosis. As their exclusion from the analysis did not essentially alter the results, we kept them in the present study.

Mammograms of the MLO view were taken using a Senographe DMR and read and recorded using a FCR AC-3CS and a CR-LP415. For the cases, the mammogram of the breast that would remain cancer-free was selected; *n*=59 for left and *n*=87 for right. For the controls, the mammogram side used for the present analysis was selected at random according to the distribution among the cases because the percentage of high-density area was somewhat higher in the left than in the right breast. Informed consent was obtained from each woman. This study was approved by the institutional review board.

The assessment of mammographic density consists of seven stages: (1) image digitalisation (0.05 mm sampling pitch and 12-bit density resolution); (2) the extraction of the breast border; (3) reduction of the image matrix; (4) the extraction of the pectoralis muscle region; (5) determination of the breast area; (6) determination of the threshold; and (7) extraction of the dense area. The details of this procedure have been described elsewhere (Matsubara *et al*, 2000). The percentage of breast density was calculated as the number of pixels within the dense area divided by the number of pixels for the entire breast area. The number of pixels was transformed into square centimeters for the total breast and dense area. The reliability of this measurement was evaluated among 38 women who revisited the screening about 1 year later. The intraclass-correlation coefficients comparing the repeated mammograms were 0.96 for total breast area and 0.90 for percent density. To evaluate the validity, our mammogram measurements were compared with those assessed by researchers at the Cancer Research Center of Hawaii (Maskarinec *et al*, 2002). They adopted the validated method described by Byng *et al* (1994) and Ursin *et al* (1998). Based on mammograms from 131 women, the rank correlation coefficients between their method and our method were 0.95 for total breast area and 0.80 for percent density.

We categorised the percent of density into five levels according to a study reported by Byrne *et al*(1995). As only one case was in the highest category, the number of categories was reduced to four for postmenopausal women. The sizes of total breast and dense area were categorised into quartile or five levels based on the distribution among the controls. Unconditional logistic regression models were applied to compute odds ratios (ORs) and 95% confidence intervals (CIs) for the categorised breast and density areas. Several known risk factors for breast cancer such as age, body mass index, age at menarche, age at first birth, number of full births, use of hormone replacement therapy, history of breast feeding, and family history of breast cancer among first-degree relatives (plus type of menopause and age at menopause in the postmenopausal group) were included in the models as covariates. The analyses were carried out separately for premenopausal and postmenopausal women. Tests for a linear trend in the logit of risk were based on continuous values. All statistical analyses were performed using SAS programs (SAS Institute, SAS/STAT user’s guide, Version 8.2, Cary, NC, USA).

## RESULTS

Table 1 shows the distributions of potential risk factors for breast cancer in the cases and the controls according to their menopausal status. In premenopausal women, the cases had significantly more years of education and had first given birth at an older age than the controls. In postmenopausal women, the cases were significantly older and had fewer births than the controls.

The unadjusted means and 95% CIs of mammographic measures are shown in Table 2. The mean of dense area of premenopausal women was significantly greater in the cases than in the controls. The percent density was higher in the cases than in the controls in premenopausal women, but the difference was of borderline significance (*P*=0.06). In postmenopausal women, the total breast area was significantly greater in the cases.

Significantly increased ORs of breast cancer were observed for breast densities of 25–49 and 50–74% in premenopausal women after controlling for age, body mass index (BMI), years of education, parity, age at menarche, lactation (yes or no), age at first birth, smoking status, alcohol intake, use of hormone replacement therapy, and family history of breast cancer among first-degree relatives (Table 3). An increase in OR for the highest category of breast density was not statistically significant in premenopausal women. A similar tendency was observed for the association between dense area and risk of breast cancer. Total breast area was not associated with risk of breast cancer in premenopausal women.

Odds ratios were significantly increased for breast densities of 25–49 and 50–100% in postmenopausal women after controlling for the covariates including age at menopause and type of menopause. The linear trend for the risk increase with increasing percent of density was statistically significant. Dense area was significantly and positively associated with the risk of breast cancer. Increasing total breast area was also significantly associated with the risk of breast cancer. Total breast area and the percent of density were independently significantly associated with the risk of breast cancer after mutual adjustment; the ORs were 1.09, 3.37, and 4.21 for breast densities of 0, 1–25, 25–49, and ⩾50% respectively, after controlling for total breast area. (The increase in risk was 2.5% for every 1% increase of percent density, *P*=0.004.) The ORs were 1.63, 3.22, and 3.43 for the second to the fourth quartile of total breast area, respectively (*P* for trend=0.005) after controlling for the percent density.

We reanalysed data after excluding 20 cases detected by the screening. The risk increase for every 1% increase of percent density was slightly decreased in premenopausal women (0.3%, *P*=0.58) and unchanged in postmenopausal women (2.3%, *P*=0.007) after controlling for the covariates. We also analysed data restricting cases to those who were detected by the screening or who had attended a mammographic screening before (*n*=55). The risk increase for every 1% increase of percent density was somewhat increased in premenopausal women (1.9%, *P*=0.03) and unchanged in postmenopausal women (2.3%, *P*=0.15). The association of total breast area with risk of breast cancer in postmenopausal women was unchanged; the OR for the highest *vs* the lowest quartile was 4.52.

## DISCUSSION

Most of the previous studies in which quantitative methods were used to assess the breast density showed ORs of 4.0 or greater for the densest categories compared with the least dense categories (Harvey and Bovbjerg, 2003). The results from the present study confirmed that breast density is an important determinant of breast cancer risk in Japanese women. However, we could not obtain a significantly increased OR for percent density of 75–100% in premenopausal women. A small number of cases included in the highest category of percent density may have resulted in the lack of association. The OR for the combined category of percent density of 50–100% was 3.08 (95% CI 0.89–10.7) after controlling for all the covariates. The value was similar to those reported in other studies (Boyd *et al*, 1982; Brisson *et al*, 1984; Van Gills *et al*, 1999). Maskarinec and Meng (2000) reported associations of percent density with breast cancer risk in ethnic groups in Hawaii. A relatively low OR (=1.4 for ⩾50% *vs* <10%) was observed for Asian women including Chinese, Filipinos, and Japanese, but a similarly low OR (=1.8) was also noted for Caucasian women in their study.

Potential sources of bias must be considered when evaluating the findings of the present study. The cases and the controls were not retrieved from the same population in the current study; therefore, selection bias may have affected the results. The controls were selected from participants in a mammographic screening. The use of cases who were detected by the same screening system was desirable, but such subjects were few. It is likely that the characteristics of the participants in the screening differ from those of the general population. If the participants in the screening were more likely to have a lower percent density than nonparticipants, the observed ORs would be overestimated. We tried to evaluate the effect of this selection bias. The use of information about the history of participation in mammographic screenings revealed that the amount of previous participation was unrelated to the breast density among the controls. We also reanalysed the data after excluding the cases detected by the screening or restricting cases to those who had previously attended mammograhic screenings. The results were not changed greatly in the reanalyses.

We observed a significant positive association between total breast area and breast cancer risk among postmenopausal women. It is not likely that the control women attended the breast cancer screening because of their small breast size. Large breast size has been suggested to be associated with the risk of breast cancer but has never been confirmed (Hsieh and Trichopoulos, 1991). Obesity has been associated with the risk of breast cancer in postmenopausal women, probably due to greater exposure of mammary epithelial tissue to endogenous oestradiol (Key *et al*, 2003). Adipose tissue in breasts may be implicated in breast cancer risk, but an adjustment for BMI should attenuate such a relationship. However, in a population with a low and homogeneous BMI, like our study subjects, the attenuation might not be great.

In this conventional case–control study, the effects of masking (Eagan and Mosteller, 1977) are different from cohort studies. If masking of cancer by breast density leads to the under-diagnosis of breast cancer, cases with high breast density will be under-represented among the diagnosed cases. On the other hand, a control subject with undetected cancer is more likely in those with high breast density. Therefore, the observed associations between breast density and the risk of breast cancer may be underestimated (Boyd *et al*, 1998).

We used mammograms taken in the MLO direction because the screening system in Japan has adopted the MLO view. Most of the previous studies conducted in other countries have used the cranio caudal (CC) direction. A high correlation between the MLO and CC views for dense area was reported by Byng *et al* (1996), although the estimates of breast density from the MLO view are systematically lower than those from the CC view. The relatively small proportion of women with high density in the present study may be due to the use of the MLO view.

We used analog films instead of digital films for 12 cases. Exclusion of these cases did not alter the results substantially; the risk increases were 0.5 and 2.1% for every 1% increase of percent density for pre- and postmenopausal women, respectively. Jeffreys *et al* (2003) reported that a digital image was more likely to be assigned a higher density than an image from film, although the magnitude of this difference was small. Therefore, it is unlikely that the observed ORs were overestimated due to the use of films for some cases.

As is the nature of retrospective design, the mammograms used in this present study were taken at the time of diagnosis. Although we used mammograms of the contra lateral breast, an underlying illness may have affected the density of the lateral breast.

The present results show that the mammographic density measured by a quantitative assessment method was associated with the risk of breast cancer in Japanese women. The possibility of risk increase in those with a larger breast size or with a high percent of density, such as >75%, should be examined in future studies.

## Figures and Tables

**Table 1:** Characteristics of cases and controls according to menopausal status^a^

	**Premenopausal women**		**Postmenopausal women**	
**Variables**	**Cases, *n*=71**	**Controls, *n*=370**	**Cases, *n***=**75**	**Controls, *n*=289**
Age (years)	43.1±6.2	42.7±5.9	62.2±9.1	58.1±6.3
BMI (kg m^−2^)	22.0±2.5	21.8±2.9	23.9±2.9	23.2±3.2
Education (years)	13.2±2.1	12.7±1.8	11.0±2.1	11.4±1.9
Age at menarch (years)	12.7±1.2	12.8±1.3	14.5±3.5	14.1±1.7
Age at first birth (years)	26.5±3.6	25.3±2.7	24.8±3.0	25.0±3.2
Age at menopause (years)	—	—	48.6±4.2	49.2±4.1
Number of parity	1.6±1.1	2.2±0.8	2.0±0.9	2.3±0.8
Alcohol intake (ml day^−1^)	7.7±17.1	6.8±18.2	5.3±12.1	4.8±13.0
Exercise (METs·h week^−1^)	6.3±6.2	4.5±5.8	4.5±7.4	4.3±7.1
Current smokers (%)	15.5	9.0	5.5	4.0
Ex-smokers (%)	4.2	5.5	4.1	3.3
Current use of HRT (%)	5.9	3.0	2.7	2.6
Past use of HRT (%)	7.4	8.3	9.5	7.7
Family history^b^ (%)	9.9	4.3	8.0	3.8
Breast feeding (%)	73.2	90.7	90.0	90.4

a Values are means±s.d. or percentage.

b Among first-degree relatives.

MET=metabolic equivalents; HRT=hormone replacement therapy.

**Table 2:** Means of mammographic measures in cases and controls according to menopausal status^a^

	**Premenopausal women**		**Postmenopausal women**	
**Variables**	**Cases**	**Controls**	**Cases**	**Controls**
Percent density (%)	45.3 (39.3–51.3)	38.2 (35.2–41.2)	18.2 (13.3–23.2)	16.2 (13.8–18.7)
Dense area (cm^2^)	27.9 (22.6–33.8)	18.9 (16.7–21.3)	8.6 (5.2–12.9)	5.8 (4.6–7.2)
Total breast area (cm^2^)	71.7 (65.7–77.9)	68.3 (65.6–71.1)	95.5 (88.7–102.6)	76.6 (73.3–80.0)

a Values are means (95% CI).

**Table 3:** Association between mammographic measures and breast cancer according to menopausal status

	**Premenopausal women**				**Postmenopausal women**		
**Variables**	**No. of cases/controls**	**Age & BMI adjusted OR (95% CI)**	**Adjusted^a^ OR (95% CI)**		**No. of cases/controls**	**Age & BMI adjusted OR (95% CI)**	**Adjusted^b^ OR (95% CI)**
*Percent density (%)*							
0	5/52	1.00	1.00	0	30/115	1.00	1.00
1–24	13/100	1.75 (0.53–5.76)	2.27 (0.64–8.08)	1–24	21/96	1.16 (0.0–2.25)	1.17 (0.55–2.49)
25–49	20/78	3.49 (1.10–11.1)	4.01 (1.16–13.9)	25–49		2.06 (0.93–4,55)	3.00 (1.20–7.48)
50–75	24/82	4.12 (1.28–13.3)	4.37 (1.24–15.4)	50–100	10/23	3.41 (1.32–8.77)	4.19 (1.33–13.2)
75–100	9/58	2.00 (0.53–7.58)	1.36 (0.31–6.06)	Every 1% (*P* for trend)		1.019 (0.007)	1.023 (0.005)
Every 1% (*P* for trend)		1.010 (0.049)	1.007 (0.22)				
							
*Dense area (cm*^*2*^)							
0	5/52	1.00		0	30/115		1.00
0.1–12.0	7/79	1.22 (0.33–4.50)	1.58 (0.41–6.23)	0.1–9.5	9/58	0.78 (0.33–1.82)	0.83 (0.33–2.12)
12.1–26.3	18/80	3.14 (0.97–10.1)	4.03 (1.14–14.2)	9.6–21.3	10/57	1.21 (0.52–2.81)	1.07 (0.41–2.80)
26.4–44.4	24/79	4.34 (1.35–14.0)	5.14 (1.45–18.3)	21.4+	26/59	3.04 (1.57–6.06)	4.02 (1.80–8.94)
44.5+	17/80	2.84 (0.87–9.30)	2.78 (0.77–10.1)	*P* for trend		0.0004	0.0002
	*P* for trend	0.02	0.09				
							
*Total breast area (cm*^*2*^)							
0–52.2	17/92	1.00	1.00	0–57.6	6/75	1.00	1.00
52.3–66.0	12/93	0.74 (0.33–1.65)	0.66 (0.28–1.56)	57.7–73.7	12/66	2.25 (0.78–6.49)	1.89 (0.61–5.91)
66.1–83.8	18/92	1.04 (0.48–2.27)	0.85 (0.36–2.04)	73.8–97.0	23/70	3.61 (1.33–9.83)	4.15 (1.39–12.4)
83.9+	24/93	1.71 (0.78–3.73)	1.53 (0.64–3.65)	97.1+	34/78	4.57 (1.63–12.8)	4.65 (1.50–14.4)
	*P* for trend	0.27	0.59	*P* for trend		0.005	0.005

a Adjusted for age, BMI, age at menarche, age at first birth, number of full births, use of hormone replacement therapy, history of breast feeding, and family history of breast cancer among first-degree relatives.

b Above, plus type of menopause (natural/surgical) and age at menopause.
